# Point-of-care Ultrasound Diagnosis of Pulmonary Embolism with Thrombus in Transit

**DOI:** 10.5811/cpcem.2018.11.40377

**Published:** 2019-01-04

**Authors:** Nicolas Kahl, Christopher Gabriel, Shadi Lahham, Maxwell Thompson, Wirachin Hoonpongsimanont

**Affiliations:** University of California, Irvine, Department of Emergency Medicine, Orange, California

## Abstract

A 95-year-old female with a history of dementia and atrial fibrillation (not on anticoagulation) presented to the emergency department (ED) by ambulance from her skilled nursing facility due to hypoxia. Point-of-care ultrasound was performed, and showed evidence of a large mobile thrombus in the right ventricle on apical four-chamber view. Further evidence of associated right heart strain was seen on the corresponding parasternal short-axis view. These ultrasound findings in combination with the patient’s clinical presentation are diagnostic of acute pulmonary embolism with right heart strain. Point-of-care transthoracic cardiac ultrasound in the ED is an effective tool to promptly diagnose acute pulmonary embolism with right heart strain and thrombus in transit and guide further treatment.

## INTRODUCTION

Deep venous thrombosis (DVT) and pulmonary embolism (PE) are dangerous conditions frequently encountered in the emergency department (ED). Clinical presentation along with diagnostic modalities, including computed tomography (CT), ultrasound and laboratory testing, may be used to arrive at the diagnosis. Thrombus in transit is defined on ultrasound as mobile echogenic material temporarily present in the right heart chambers on its way to the pulmonary circulation, and it is diagnostic of PE.^7^ We present a case report of an elderly female who was brought by ambulance to the ED in acute respiratory distress, and was immediately evaluated with point-of-care ultrasound (POCUS).

## CASE REPORT

A 95-year-old female with a history of dementia and atrial fibrillation (not on anticoagulation) presented to the ED by ambulance from her skilled nursing facility due to hypoxia. The patient had been requiring 2–4 liters of oxygen via nasal cannula at her nursing facility; however, in the ED the patient’s oxygen saturation was 80% on a non-rebreather face mask. Physical exam was notable for tachycardia, tachypnea, use of accessory muscles for respiration, and somnolence. The patient did not have clinical signs of DVT such as unilateral leg swelling or calf tenderness. Of note, the patient had an allergy to iodinated contrast. POCUS was performed and showed evidence of a large mobile thrombus in the right ventricle on apical four-chamber view (Video). Further evidence of associated right heart strain was seen in the corresponding parasternal short-axis view. There was no evidence of a pericardial effusion. These ultrasound findings in combination with the patient’s clinical presentation were diagnostic of acute PE with right heart strain.^1^,^2^ The patient’s family arrived in the ED, and her code status was established as “do not resuscitate” with comfort measures only. Further imaging, fibrinolysis and thrombectomy were not attempted in accordance with the patient’s wishes.

## DISCUSSION

DVT and associated PE is a potentially devastating problem encountered in the ED. The gold standard for diagnosis is CT angiogram. However, in order to undergo CT angiography patients must have adequate renal function and cannot have contrast allergies.^6^ A visible right heart thrombus on ultrasound is a rare finding in acute PE and is associated with a poor prognosis.^3^ The high mortality rate of 44.7% is due to the potential for imminent embolization to the pulmonary arteries, which can cause obstructive shock.^3^ Other evidence of right heart strain on ultrasound includes bowing of the intraventricular septum into the left ventricle, right ventricular systolic dysfunction, and McConnell’s sign. McConnell’s sign is the most specific finding at 94% and is defined as right ventricular free wall akinesis with sparing of the apex.^4^ POCUS allows for rapid diagnostic assessment that can guide therapy for time-sensitive, critically ill patients. Furthermore, in patients with contraindications to iodinated contrast, ultrasound is an acceptable alternative to CT angiogram. Our patient’s allergy to iodine made ultrasound preferable to CT angiogram. Immediate thrombolysis or surgical embolectomy to prevent circulatory collapse should be considered in these high-risk patients with signs of right ventricular dysfunction and visible thombi in transit.^5^

## CONCLUSION

Point-of-care transthoracic cardiac ultrasound in the ED is an effective tool to promptly diagnose acute pulmonary embolism with right heart strain and thrombus in transit and guide further treatment. Our patient was 95 years old and wanted comfort measures only, but the use of cardiac ultrasound could have expedited potentially life-saving fibrinolysis or thrombectomy if she had wanted it. The risk of delaying medical decision-making and treatment of acute pulmonary embolism with right heart strain makes the consideration of using rapid point-of-care transthoracic cardiac ultrasound in this setting critically important for emergency physicians.

**CPC-EM Capsule**

What do we already know about this clinical entity?

*Pulmonary embolism (PE) is frequently encountered in the emergency department. Clinical presentation and various diagnostic modalities are used to arrive at the diagnosis*.

What makes this presentation of disease reportable?

*Thrombus in transit is a rare finding on ultrasound, and has important diagnostic and therapeutic implications in management of PE*.

What is the major learning point?

*Point-of-care ultrasound (POCUS) can be used to rapidly diagnose pulmonary embolism and guide management and intervention prior to computed tomography angiogram or further diagnostic testing*.

How might this improve emergency medicine practice?

*Emergency physicians can consider POCUS as a first-line diagnostic modality in a patient who presents with clinical concern for PE*.

## Supplementary Information

Video Apical four-chamber view of the heart shows mobile thrombus in transit between the right atrium and right ventricle. There is right atrial dilation and evidence of McConnell’s sign.
